# Propofol protects against high glucose-induced endothelial adhesion molecules expression in human umbilical vein endothelial cells

**DOI:** 10.1186/1475-2840-12-13

**Published:** 2013-01-11

**Authors:** Minmin Zhu, Jiawei Chen, Hui Jiang, Changhong Miao

**Affiliations:** 1 Department of Anaesthesiology and Oncology, Shanghai Medical College, Fudan University Shanghai Cancer Centre, Shanghai, People’s Republic of China

**Keywords:** Propofol, High glucose, Adhesion molecules, NF-κB, HUVECs

## Abstract

**Background:**

Hyperglycemia could induce oxidative stress, activate transcription factor nuclear factor kappa B (NF-κB), up-regulate expression of endothelial adhesion molecules, and lead to endothelial injury. Studies have indicated that propofol could attenuate oxidative stress and suppress NF-κB activation in some situations. In the present study, we examined whether and how propofol improved high glucose-induced up-regulation of endothelial adhesion molecules in human umbilical vein endothelial cells (HUVECs).

**Methods:**

Protein expression of endothelial adhesion molecules, NF-κB, inhibitory subunit of NF-κBα (IκBα), protein kinase Cβ2 (PKCβ2), and phosphorylation of PKCβ2 (Ser^660^) were measured by Western blot. NF-κB activity was measured by electrophoretic mobility shift assay. PKC activity was measured with SignaTECT PKC assay system. Superoxide anion (O_2_^.-^) accumulation was measured with the reduction of ferricytochrome c assay. Human peripheral mononuclear cells were prepared with Histopaque-1077 solution.

**Results:**

High glucose induced the expression of endothelial selectin (E-selectin), intercellular adhesion molecule 1 (ICAM-1), vascular cell adhesion molecule 1 (VCAM-1), and increased mononuclear-endothelial adhesion. High glucose induced O_2_^.-^ accumulation, PKCβ2 phosphorylation and PKC activation. Further, high glucose decreased IκBα expression in cytoplasm, increased the translocation of NF-κB from cytoplasm to nuclear, and induced NF-κB activation. Importantly, we found these high glucose-mediated effects were attenuated by propofol pretreatment. Moreover, CGP53353, a selective PKCβ2 inhibitor, decreased high glucose-induced NF-κB activation, adhesion molecules expression, and mononuclear-endothelial adhesion.

**Conclusion:**

Propofol, via decreasing O_2_^.-^ accumulation, down-regulating PKCβ2 Ser^660^ phosphorylation and PKC as well as NF-κB activity, attenuated high glucose-induced endothelial adhesion molecules expression and mononuclear-endothelial adhesion.

## Background

Perioperative hyperglycemia, a metabolic alteration caused by perioperative physiological stress and excessive glucose infusion, was commonly seen in non-diabetics [1] as well as diabetics [2]. Hyperglycemia could up-regulate the expression of endothelial adhesion molecules, such as endothelial selectin (E-selectin), intercellular adhesion molecule 1 (ICAM-1), and vascular cell adhesion molecule 1 (VCAM-1) [3-5], thus augmenting pathological leukocytes-endothelial adhesion [3] and leading to endothelial dysfunction and injury. Accordingly, during perioperative period, especially for hyperglycemic patients, interventions that may inhibit endothelial adhesion molecules expression are potential strategies to protect endothelial cells from dysfunction and its sequelae.

Hyperglycemia could induce overproduction of reactive oxygen species (ROS), which is one of the major factors responsible for diabetic vascular injury [6,7]. The nuclear factor kappa B (NF-κB) signal pathway was also reported to be involved in high glucose-induced up-regulation of endothelial adhesion molecules [3,8]. Lee et al. and Kwon et al. reported that high glucose-induced up-regulation of endothelial adhesion molecules was alleviated by inhibiting ROS generation and NF-κB activity [8,9]. Propofol (2, 6-diisopropylphenol) is a widely used intravenous anesthetic agent. Chen J et al. found propofol could suppress oxidative stress, NF-κB activation and mononuclear-endothelial adhesion in endothelial cells exposed to hydrogen peroxide [10]. However, the protective effects of propofol on hyperglycemia-induced expression of endothelial adhesion molecules have not been well studied. In the present study, we examined whether propofol improved high glucose-induced up-regulation of endothelial adhesion molecules and mononuclear-endothelial adhesion in human umbilical vein endothelial cells (HUVECs). More importantly, we explored the potential underlying mechanisms.

## Methods

### Cell culture

HUVECs (Clonetics) were grown in Dulbecco’s modified Eagle medium (DMEM) with 5 mM glucose and 10% fetal bovine serum. Cells were cultured in an incubator supplemented with 5% CO_2_-95% air at 37°C and sub-cultured on reaching 90% confluence. The fourth passage of cells was employed in this study.

### Study design

HUVECs were cultured with different concentrations (5, 10, 15, 20 and 30 mM) of glucose for different times (1, 2, 4 and 8 h). By measuring adhesion molecules expression, we determined the appropriate glucose treatment condition with maximal effect on endothelial adhesion molecules expression. During general anesthesia, plasma concentrations of propofol range from 5 to 50uM [11]. To mimic *in vivo* situations, HUVECs were pre-incubated with different concentrations (5, 10, 20, and 40uM) of propofol (Sigma) for 30 min, followed by glucose treatment. The optimal concentration of propofol with significant inhibitory effects on endothelial adhesion molecules expression was determined. These treatment conditions were used in the subsequent studies in which HUVECs were cultured and divided into four groups to examine the underlying signaling pathways. Group 1: HUVECs were cultured in 5 mM glucose for 4 h as control; Group 2: HUVECs were pre-incubated with 10uM propofol for 30 min and treated with 5 mM glucose for 4 h; Group 3: HUVECs were treated with 15 mM glucose for 4 h; Group 4: HUVECs were pre-incubated with 10uM propofol for 30 min and treated with 15 mM glucose for 4 h.

### Western blot analysis

Whole-cell extracts, nuclear extracts and cytoplasmic extracts were prepared with the use of Nuclear Extract Kit (Active Motif) according to the manufacturer’s instructions. Equal amounts of protein were separated by 6% or 8% SDS-PAGE and transferred to PVDF membranes. After being blocked in 5% skim milk, the membranes were incubated with an appropriate dilution of primary antibody at 4°C for overnight. The primary antibodies were monoclonal antibody against E-selectin (Santa Cruz Biotechnology), protein kinase Cβ2 (PKCβ2) (Abcam), Phospho-PKCβ2(ser^660^)( Abcam), β-actin (Santa Cruz Biotechnology), and polyclonal antibody against ICAM-1 (Cell Signaling Technology),VCAM-1 (Santa Cruz Biotechnology), NF-κB p65 (Cell Signaling Technology), inhibitory subunit of nuclear factor-κBα (IκBα) (Santa Cruz Biotechnology), Histone H3 (Santa Cruz Biotechnology). Then, the membranes were incubated with secondary antibody, washed, and detected with the ECL system. The relative densities of protein bands were analyzed by Scan-gel-it software.

### PKC activity assay

PKC activity was measured with SignaTECT PKC assay system (Promega) according to the manufacturer’s instructions. In brief, membrane extracts, reaction buffer (100 mM Tris–HCl, 1.6 mg/ml phosphatidylserine, 0.16 mg/ml diacylglycerol, 50 mM MgCl2) and [γ-^32^P] ATP were mixed and kept at 30°C for 10 min. PKC phosphorylation was determined by measuring the radioactivity. The enzymatic activity of PKC was determined by subtracting the activity of the enzyme in the absence of phospholipids from that of the enzyme in the presence of phospholipids. The results were shown as folds increased compared with 5 mM glucose group.

### Electrophoretic mobility shift assay

NF-κB activity was measured by electrophoretic mobility shift assay as described previously [10]. In brief, [γ-^32^P] ATP (Amersham Life Sciences) was used to end-label the complementary oligonucleotides containing NF-κB binding site (5’-TGTCGAATGCAAATCACTAGAA-3’), and QIAquick Nucleotide Removal Kit (Qiagene) was use to eliminate the unincorporated [γ-^32^P] ATP. The radiolabeled probes and nuclear extracts were incubated together for 30 min. For supershift assay, 5 μg rabbit polyclonal antibody against NF-κB (Santa Cruz Biotechnology) was mixed with nuclear extracts on ice for 1 h before the addition of radiolabeled probes. The specificity was verified by supershift assay with 5 μg normal rabbit IgG (Santa Cruz Biotechnology). For competition assay, 100-fold excess of nonradiolabeled probes were incubated with nuclear extracts for 10 min before the addition of radiolabeled probes. As negative control for competition assay, 100-fold excess of nonradiolabeled probes containing a mutant NF-κB binding site (5’-TGTCGAATGCAAGCCACTAG AA-3’) were used as competitors. The DNA-protein complexes were separated by electrophoresis in a 6% non-denaturing polyacrylamide gel. The gels were dried for 1 h, and exposed to a radiographic film for 24 h at −80°C.

### Superoxide anion (O_2_^.-^ ) accumulation assay

O_2_^.-^ accumulation was measured by the reduction of ferricytochrome c assay as described previously [12]. Briefly, HUVECs were washed and cultured with Krebs-HEPES buffer containing 20 μM ferricytochrome c (Sigma) with or without superoxide dismutase (Sigma). The absorbance was read spectrophotometrically at 550 nm. Reduction of ferricytochrome c with superoxide dismutase was subtracted from the values without superoxide dismutase.

### Isolation and adhesion of mononuclear cells to HUVECs

The isolation of human peripheral mononuclear cells was prepared with the use of Histopaque-1077 (Sigma). In brief, 5 ml heparinized blood from healthy volunteers was carefully layered onto 5 ml Histopaque-1077. The mononuclear cells were collected after the blood was centrifuged at 400 g for 30 min. Isolated mononuclear cells were then washed twice with PBS, suspended in the culture medium, and added to the HUVECs . After incubation at 37°C for 30 min, cells were washed three times with PBS, and observed under a phase-contrast microscope. Adherent cells were counted in 10 different fields from three separate culture dishes. Written informed consent was obtained from the volunteers for publication of this report and any accompanying images.

### Statistical analysis

Data were expressed as mean ± SD, and n indicates number of experiments. Statistical significance was determined in multiple comparisons among independent groups of data in which analysis of variance indicated the presence of significant difference. A value of p < 0.05 was considered significant.

## Results

### 15 mM glucose-induced endothelial adhesion molecules expression and its modulation by propofol

**Figure 1:**
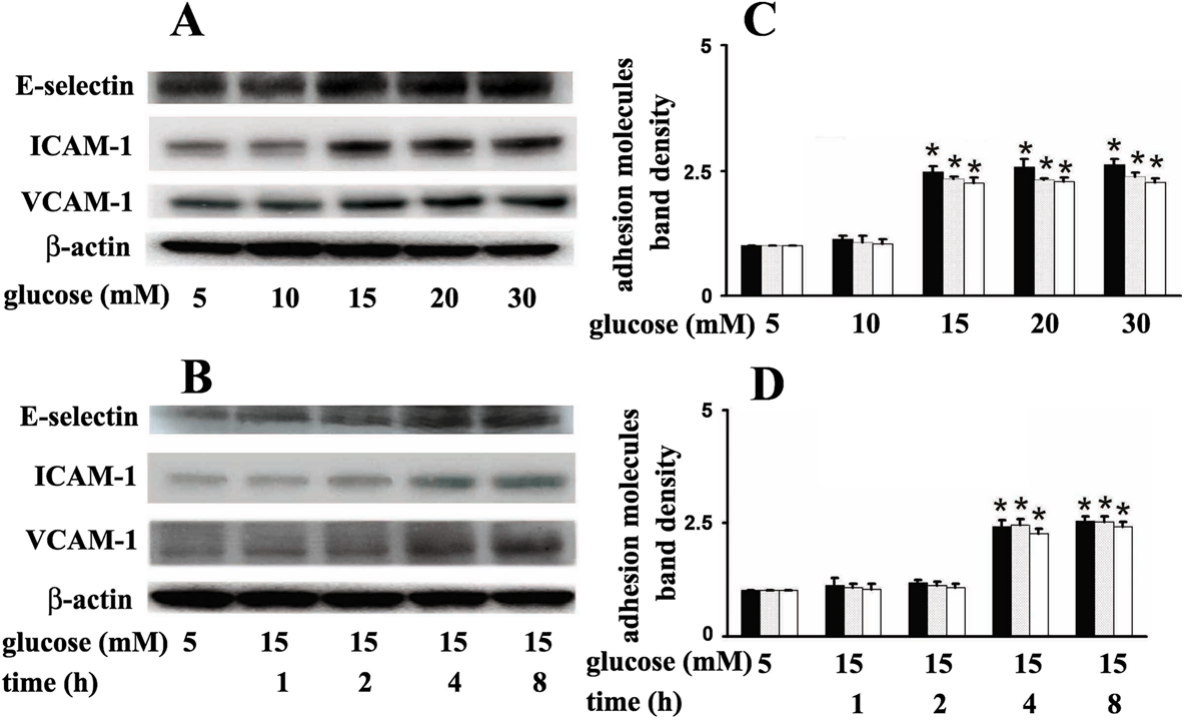
**Effects of glucose on adhesion molecules expression in human umbilical vein endothelial cells (HUVECs).(A)** HUVECs were cultured in different concentrations of glucose (5, 10, 15, 20, 30 mM) for 4 hours. **(B)** HUVECs were cultured in 5 mM or 15 mM glucose for different times (1, 2, 4, 8 h). **(C, D)** Quantification of protein band density of adhesion molecules (*p < 0.05 vs. 5 mM glucose, n = 5). Data were expressed as mean ± SD. ■ represents E-selectin, ░ represents ICAM-1, □ represents VCAM-1.

**Figure 2:**
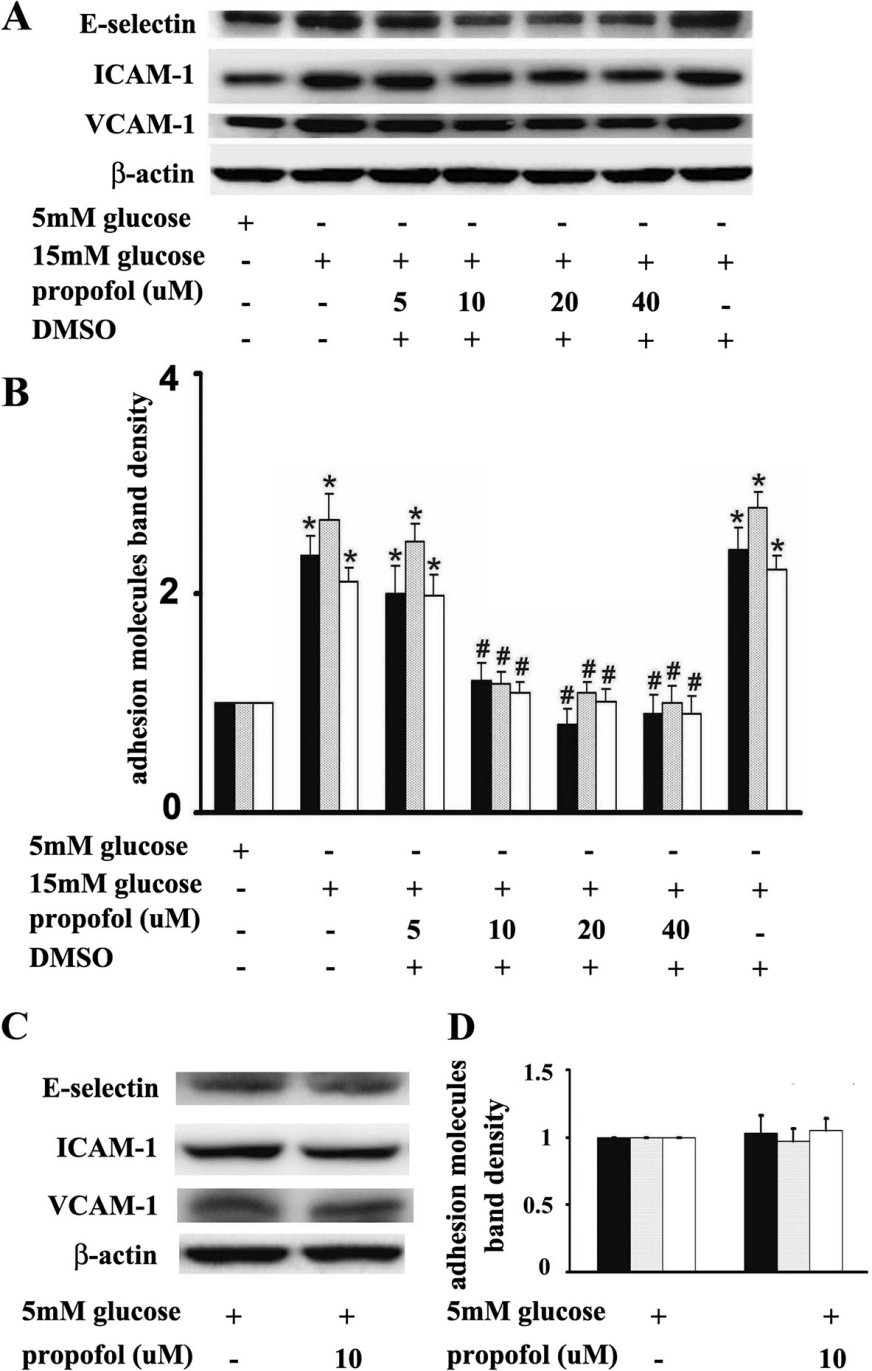
**Effects of propofol on adhesion molecules expression in hyperglycemic human umbilical vein endothelial cells (HUVECs).(A)** HUVECs were pre-incubated with different concentrations (5, 10, 20, and 40uM) of propofol for 30 min, followed by glucose treatment. **(B)** Quantification of protein band density of adhesion molecules (*p < 0.05 vs. 5 mM glucose, #p < 0.05 vs. 15 mM glucose, n = 5). **(C)** HUVECs were pre-incubated with 10uM propofol for 30 min, followed by 5 mM glucose treatment. **(D)** Quantification of protein band density of adhesion molecules (*p < 0.05 vs. 5 mM glucose, #p < 0.05 vs. 15 mM glucose, n = 5). Data were expressed as mean ± SD. ■ represents E-selectin, ░ represents ICAM-1, □ represents VCAM-1.

In HUVECs, compared with 5 mM glucose treatment, high concentrations of glucose caused a marked up-regulation of adhesion molecules expression in a concentration- and time-dependent manner. Incubation of cells with 15 mM glucose for 4 h caused a significant up-regulation of E-selectin, ICAM-1 and VCAM-1 expression (Figure 1). We also found that propofol pre-treatment could attenuate 15 mM glucose-induced adhesion molecules expression in a concentration-dependent manner. Compared with 15 mM glucose treatment, pre-incubation of cells with 10uM propofol for 30 min significantly attenuated 15 mM glucose-induced adhesion molecules expression (Figure 2A, B). Propofol solvent dimethyl sulfoxide (DMSO) did not affect 15 mM glucose-induced adhesion molecules expression (Figure 2A, B).

Please note pre-incubation of cells with 10uM propofol for 30 min had no effect on endothelial adhesion molecules expression in cells cultured in 5 mM glucose (Figure 2C, D).

### 15 mM glucose-induced NF-κB, PKC and PKCβ2 activation and its modulation by propofol

**Figure 3:**
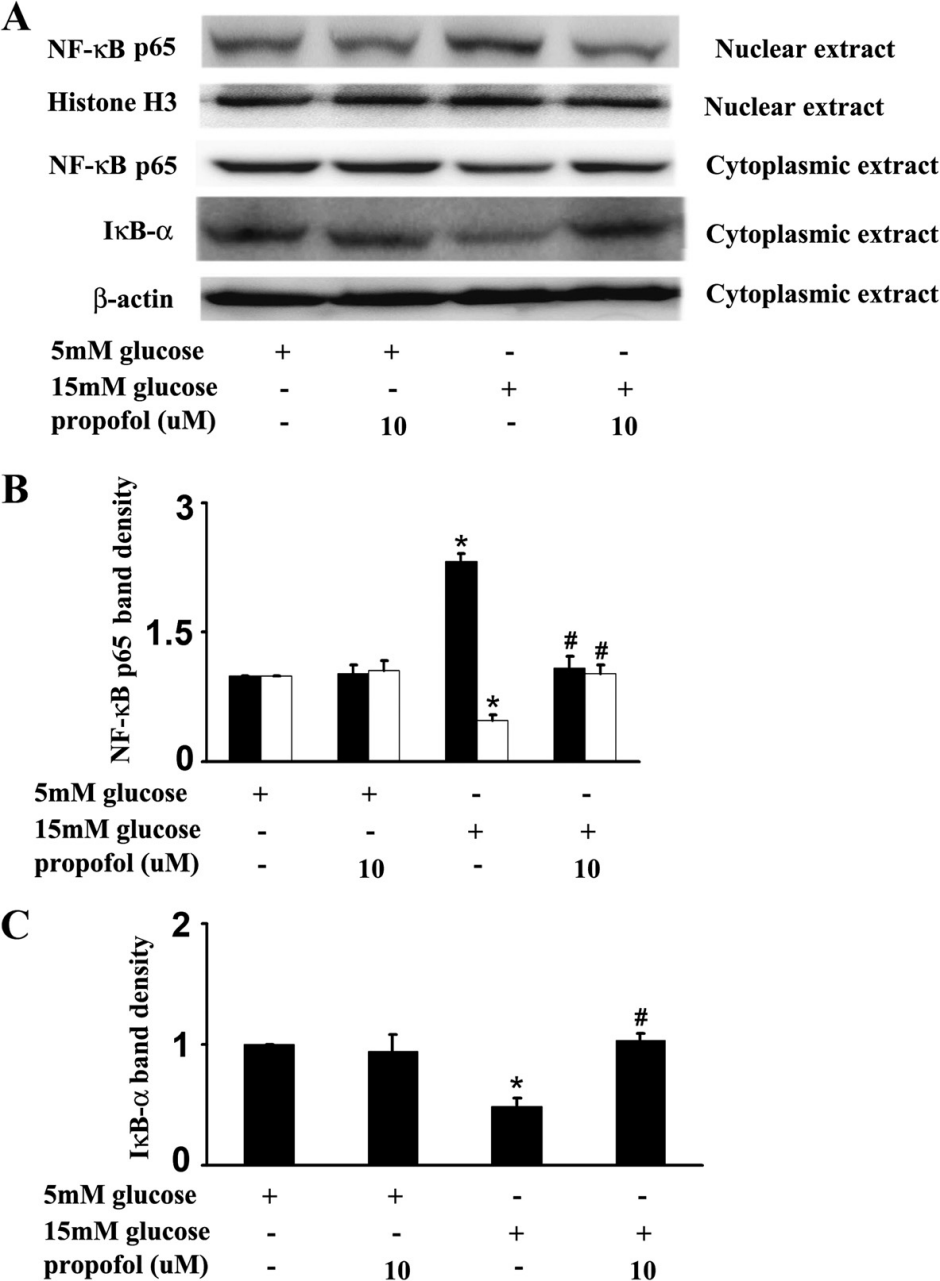
**Effects of propofol on 15 mM glucose-mediated nuclear factor kappa B (NF-κB) signal pathway.(A)** Human umbilical vein endothelial cells were cultured in either 5 mM glucose or 15 mM glucose for 4 h with corresponding treatment. **(B)** Quantification of protein band density of NF-κB (p65) (*p < 0.05 vs. 5 mM glucose, #p < 0.05 vs. 15 mM glucose, n = 5). ■ represents nuclear extract, □ represents cytoplasmic extract. **(C)** Quantification of protein band density of IκBα (*p < 0.05 vs. 5 mM glucose, #p < 0.05 vs. 15 mM glucose, n = 5). Data were shown as mean ± SD.

**Figure 4:**
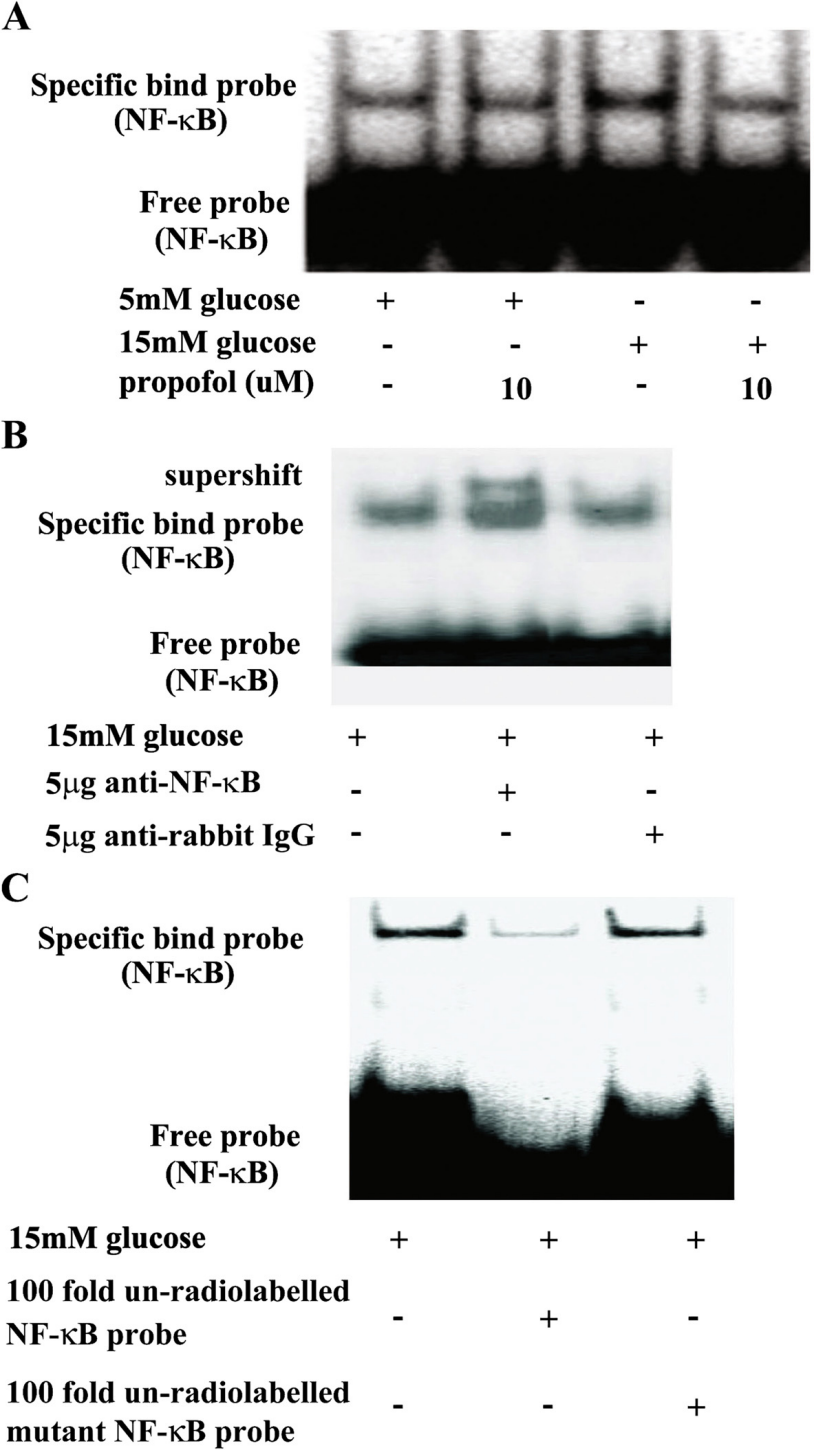
**Effects of propofol on 15 mM glucose-induced nuclear factor kappa B (NF-κB) activation. (A)** Human umbilical vein endothelial cells were cultured in either 5 mM glucose or 15 mM glucose for 4 h with corresponding treatment. Shifted band was increased by 15 mM glucose, but reduced by propofol (n = 5). **(B)** In the supershift assay, 15 mM glucose-induced band was shifted by the pre-incubation of nuclear extracts with anti-NF-κB antibody, but was not affected by normal rabbit IgG (n = 5). **(C)** In the competition assay, 100-fold excess of nonradiolabeled probes were incubated with nuclear extracts 10 min before the addition of radiolabeled probes. 15 mM glucose-induced shifted band was blocked by nonradiolabeled competitors, but mutant competitors had no such effect (n = 5).

In HUVECs, compared with 5 mM glucose treatment, 15 mM glucose increased the expression of NF-κB p65 protein in the nuclear extracts and decreased its expression in the cytoplasmic extracts (Figure 3A, B), and these effects were alleviated by propofol pre-treatment. Consistently, compared with 5 mM glucose treatment, 15 mM glucose deceased IκBα expression in the cytoplasmic extracts (Figure 3A, C), which was also recovered by propofol pre-treatment (Figure 3A, C). The effect of 15 mM glucose and propofol on NF-κB activity was determined by electrophoretic mobility shift assay. We found 15 mM glucose induced NF-κB activation, which was attenuated by propofol pre-treatment (Figure 4A). The specificity was confirmed by supershift assay and competition assay (Figure 4B, C).

**Figure 5:**
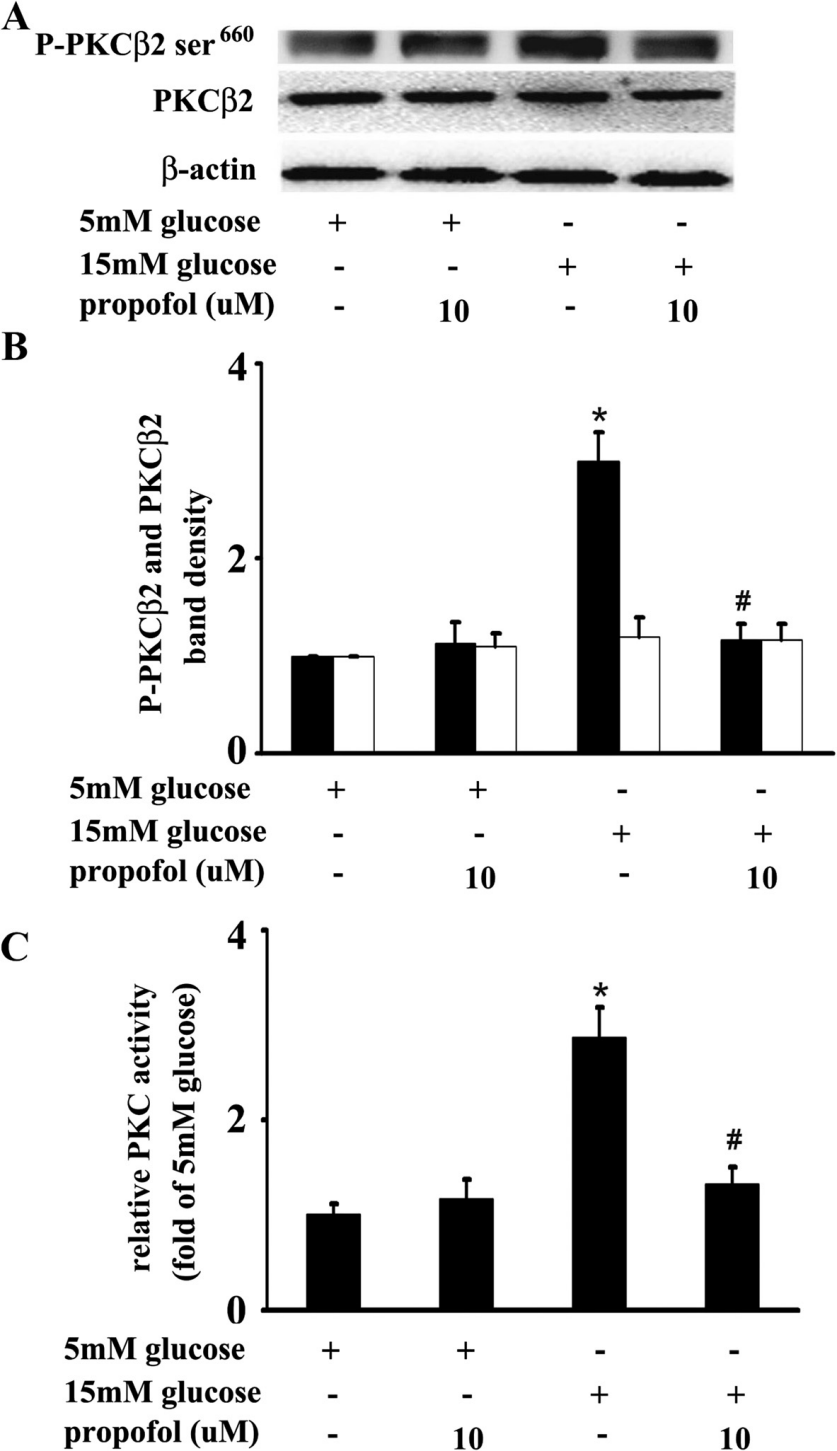
**Effects of propofol on 15 mM glucose-induced Ser**^**660**^**phosphorylation of protein kinase Cβ2 (PKCβ2) and PKC activation. (A)** Human umbilical vein endothelial cells (HUVECs) were cultured in either 5 mM glucose or 15 mM glucose for 4 h with corresponding treatment. **(B)** Quantification of protein band density of PKCβ2 or phosphorylated PKCβ2 (*p < 0.05 vs. 5 mM glucose, #p < 0.05 vs. 15 mM glucose, n = 5). ■ represents PKCβ2 phosphorylation, □ represents PKCβ2. **(C)** HUVECs were cultured in either 5 mM glucose or 15 mM glucose for 4 h with corresponding treatment. (*p < 0.05 vs. 5 mM glucose, #p < 0.05 vs. 15 mM glucose, n = 5). The results were shown as folds increased compared with 5 mM glucose group.

Compared with 5 mM glucose treatment, 15 mM glucose increased Ser^660^ phosphorylation of PKCβ2, which was attenuated by propofol. However, compared with 5 mM glucose, 15 mM glucose and propofol had no effect on PKCβ2 expression (Figure 5A, B). Moreover, compared with 5 mM glucose treatment, 15 mM glucose induced marked PKC activation, which was attenuated by propofol (Figure 5C).

### 15 mM glucose-induced O_2_^.-^ accumulation and its modulation by propofol

**Figure 6:**
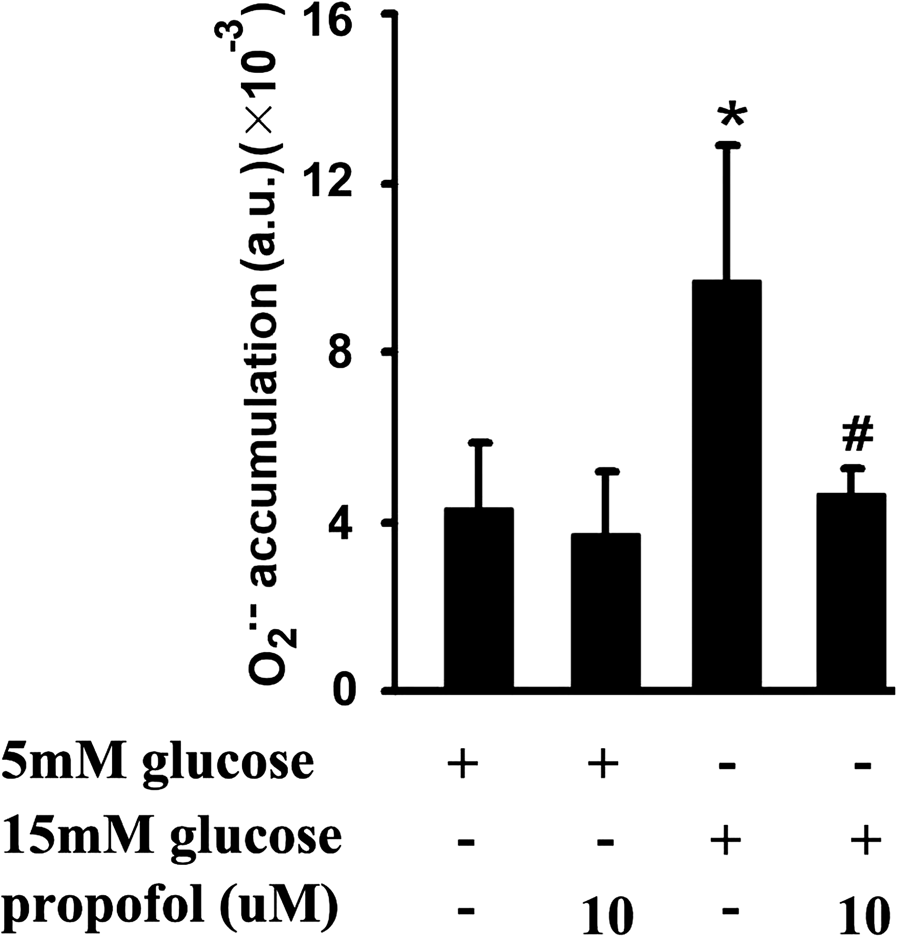
**Effects of propofol on 15 mM glucose-induced superoxide anion (O**_**2**_^**.-**^**) accumulation.** Human umbilical vein endothelial cells were cultured in either 5 mM glucose or 15 mM glucose for 4 h with corresponding treatment. (*p < 0.05 vs. 5 mM glucose, #p < 0.05 vs. 15 mM glucose, n = 5). Data were shown as mean ± SD. (a.u. = arbitrary units).

Compared with 5 mM glucose treatment, 15 mM glucose induced a significant increase of O_2_^.-^ accumulation. More importantly, we found that propofol could mitigate 15 mM glucose-induced O_2_^.-^ accumulation (Figure 6).

### 15 mM glucose-induced endothelial dysfunction and its modulation by propofol and PKCβ2 inhibitor

**Figure 7:**
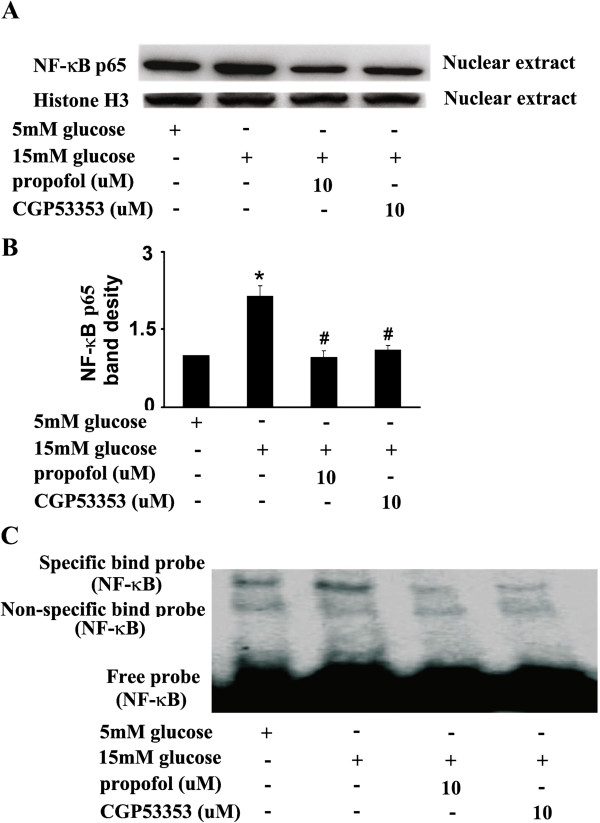
**Effects of propofol and CGP53353 on 15 mM glucose-induced nuclear factor kappa B (NF-κB) activation.(A)** Human umbilical vein endothelial cells (HUVECs) were cultured in either 5 mM glucose or 15 mM glucose for 4 h with corresponding treatment. **(B)** Quantification of protein band density of NF-κB (p65) (*p < 0.05 vs. 5 mM glucose, #p < 0.05 vs. 15 mM glucose, n = 5). Data were shown as mean ± SD. (**C**) HUVECs were cultured in either 5 mM glucose or 15 mM glucose for 4 h with corresponding treatment. Shifted band was increased by 15 mM glucose, but reduced by propofol or CGP53353 (n = 5).

CGP53353, a highly selective inhibitor of PKCβ2, could decrease 15 mM glucose-induced expression of NF-κB p65 protein in the nuclear extracts (Figure 7A, B), and the effect was similar to that of propofol. We also found that 15 mM glucose-induced NF-κB activation was attenuated by CGP53353 (Figure 7C).

**Figure 8:**
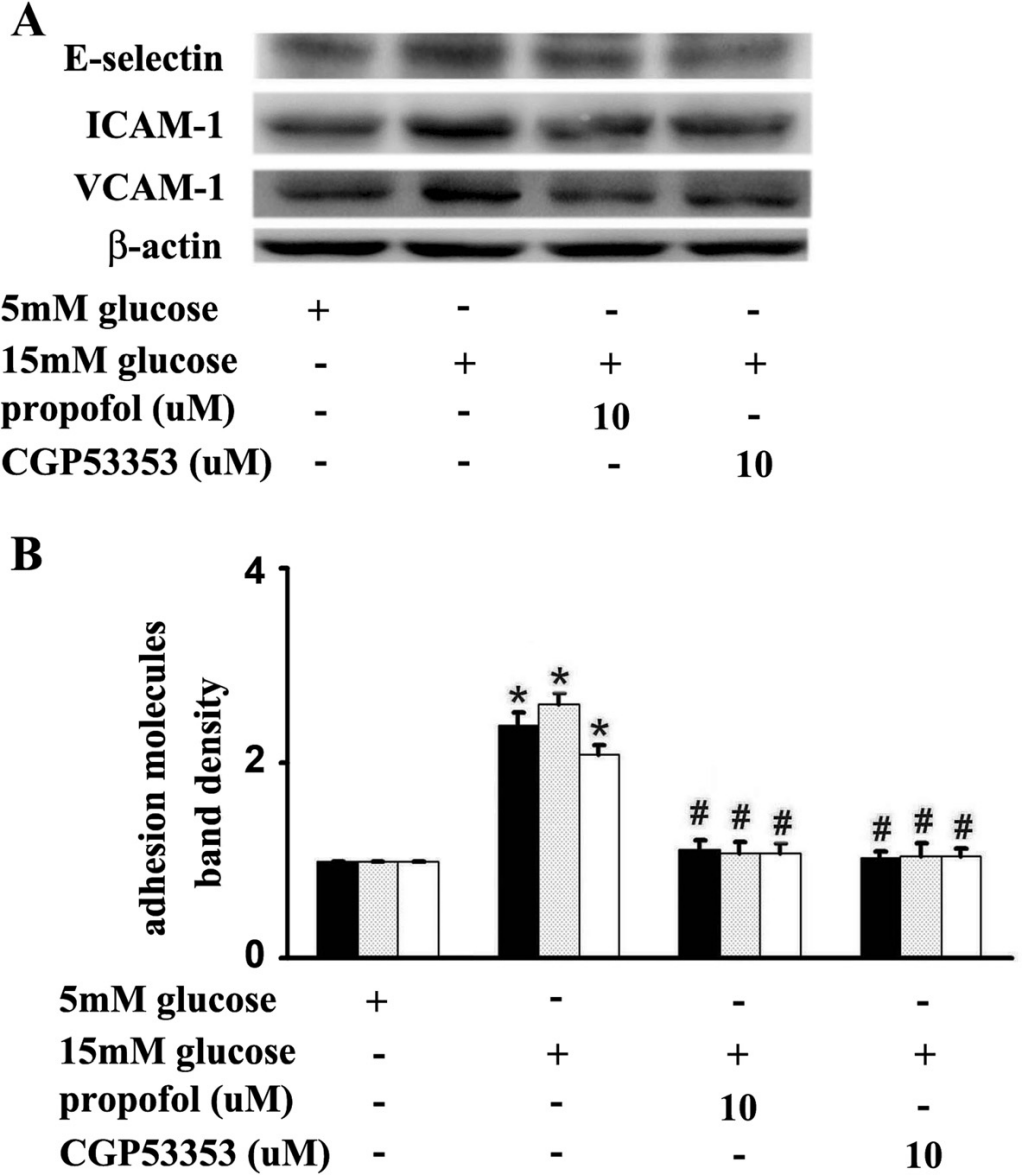
**Effects of propofol and CGP53353 on 15 mM glucose-induced adhesion molecule expression. (A)** Human umbilical vein endothelial cells were cultured in either 5 mM glucose or 15 mM glucose for 4 h with corresponding treatment. **(B)** Quantification of protein band density of adhesion molecules (*p < 0.05 vs. 5 mM glucose, #p < 0.05 vs. 15 mM glucose, n = 5). Data were shown as mean ± SD. ■ represents E-selectin, ░ represents ICAM-1, □ represents VCAM-1.

**Figure 9:**
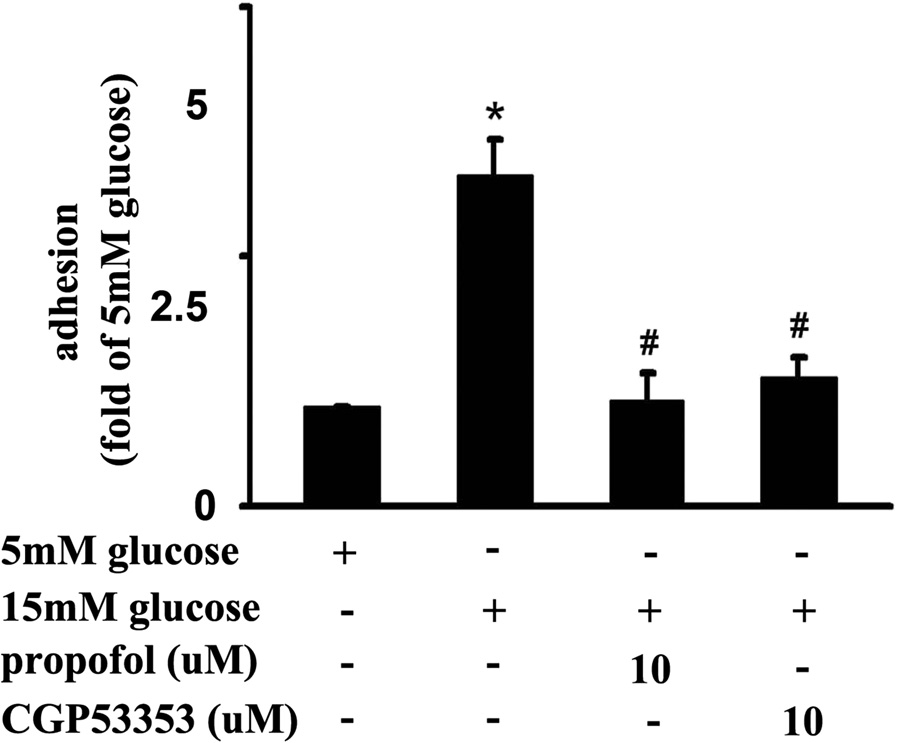
**15mM glucose-induced mononuclear-endothelial adhesion and its modulation by propofol and CGP53353.** Compared with 5 mM glucose, 15 mM glucose greatly induced mononuclear-endothelial adhesion, which was ameliorated by propofol or CGP53353 pretreatment (*p < 0.05 vs. 5 mM glucose, #p < 0.05 vs. 15 mM glucose, n = 5). Data were shown as mean ± SD.

In addition, CGP53353 and propofol decreased 15 mM glucose-induced expression of adhesion molecules to a similar extent (Figure 8). Consistently, 15 mM glucose greatly induced mononuclear-endothelial adhesion, which was attenuated by propofol or CGP53353 pre-treatment (Figure 9).

## Discussion

The main finding of the present study is that propofol could protect HUVECs against 15 mM glucose-induced endothelial adhesion molecules expression and mononuclear-endothelial interaction. Our data also suggested that the protective effects of propofol might be achieved by attenuating O_2_^.-^ accumulation, inhibiting PKCβ2 ser^660^ phosphorylation, PKC activity and NF-κB activation.

Hyperglycemia could up-regulate endothelial adhesion molecules expression, thus leading to the adhesion of monocytes to endothelial cells [3-5]. Many studies indicated that NF-κB signal pathway was involved in the hyperglycemia-mediated up-regulation of gene and protein expression of endothelial adhesion molecules, including VCAM-1, ICAM-1, and E-selectin [3-5,13-16]. These findings were consistent with our study (Figures 1, 2, 3, 4). Further, we suggested that PKCβ2 was involved in this process and its activation laid up-stream of NF-κB activation (Figure 5, 7, 8, 9). This was consistent with a previous study, which demonstrated that PKCβ2 and NF-κB played a key role in high glucose-mediated up-regulation of VCAM-1 in vascular endothelial cells [17]. However, other pathways were also claimed to be responsible. Kim S et al. reported that high glucose-induced endothelial adhesion molecules expression was mediated through the p38 mitogen-activated protein kinase (MAPK) signaling pathway [18]. In our preliminary experiments, we examined the activity of p38 MAPK, but did not detect its activation in response to 15 mM glucose (data not shown). We noticed that endothelial cells were treated with 25 mM glucose for 24 h in their study, while we treated endothelial cells with 15 mM glucose for 4 h. One potential explanation for the discrepancy between their findings and ours is that different concentration or duration of glucose treatment may up-regulate the expression of endothelial adhesion molecules through different pathways.

The necessity to regulate plasma glucose concentration to normal levels in perioperative hyperglycemic patients is debatable, because it may cause severe hypoglycemia and other serious adverse events [19]. As such, scientists are devoting to explore novel strategies, which may exert beneficial effects without tightly regulating plasma glucose levels. Recently, endothelial adhesion molecules have been widely considered to be potential targets for the effective treatment of high glucose-induced endothelial injury [20]. It has been shown that high glucose-induced mononuclear-endothelial cell adhesion and endothelial injury can be attenuated by several compounds, such as fasudil [21] and cannabidiol [22]. Interestingly, these compounds have the property to reduce superoxide generation and decrease endothelial adhesion molecules expression [21,22]. So, antioxidant may be a novel vascular protective strategy for hyperglycemic patients.

Propofol is an intravenous anesthetic agent which is widely used in clinical settings. Besides anesthetic properties, other characteristics of propofol have been widely studied in recent years. Propofol is chemically similar to endogenous antioxidant a-tocopheral (Vitamin E), and theoretically it should demonstrate similar properties [23]. Chen J et al. reported that propofol could attenuate the adhesion of monocytes to oxidative stress-activated HUVECs [10]. This was quite consistent with ours. In the present study, we found that propofol could significantly alleviate 15 mM glucose-induced O_2_^.-^ accumulation (Figure 6), endothelial adhesion molecules expression (Figure 2) and mononuclear-endothelial cell interaction (Figure 9). So we believe the antioxidative property of propofol is one of the main mechanisms for its protective effects on HUVECs.

The activation of NF-κB consists of several steps, starting with the degradation of IκB, a cytoplasmic inhibitor of NF-κB [24]. Among IκB family, IκBα is the most widely studied [25]. IκBα degradation leads to the translocation of NF-κB from the cytoplasm compartment to the nuclear, where it could recognize and bind to the promoter of target genes and regulate the expression of these genes [26]. Studies have indicated that propofol could inhibit NF-κB activation in endothelial cells exposed to hydrogen peroxide [10] and lipopolysaccharide [27]. In the present study, we found that pre-treatment of cells with propofol attenuated 15 mM glucose-induced cytoplasmic IκBα degradation and NF-κB translocation (Figure 3). In addition, we demonstrated that propofol could inhibit 15 mM glucose-induced endothelial adhesion molecules expression (Figure 2) and mononuclear-endothelial cell interaction (Figure 9). These results strongly suggested that the beneficial effects of propofol on 15 mM glucose-induced endothelial adhesion molecules expression may result from its inhibitory effects on NF-κB signal pathway.

It is of interest to determine the leading cause of PKC activation in response to high glucose. Gallo A et al., Gopalakrishna R et al. and Pricci F et al. reported that high glucose increased oxidative stress, which is responsible for the translocation and activation of PKC in vascular tissues [28-30]. It has also been reported that activation of PKCβ2 was associated with high glucose-induced vascular ROS generation [31] and high glucose-mediated nitric oxide reduction [32]. In the present study, we found propofol could attenuate 15 mM glucose-induced O_2_^.-^ accumulation (Figure 6). Consistently, our previous study showed that propofol could restore high glucose-mediated O_2_^.-^ accumulation and nitric oxide reduction via re-coupling endothelial nitric oxide synthase [33]. Accordingly, we suggested that the antioxidative effect of propofol is a potential mechanism responsible for the beneficial effect on high glucose-mediated PKC activation and mononuclear-endothelial adhesion.

In a recent study, vascular healing responses were described after drug-eluting stent implantation in experimental models, employing everolimus or paclitaxel [34]. In these models, anesthesia was achieved with propofol. We may raise the assumption that the obtained favorable effects were at least partially due to propofol. Further studies in *in vivo* models are necessary to verify this hypothesis.

This study has some limitations. First, the study was carried out in HUVECs, which is an *in vitro* system. It differs from *in vivo* settings, especially when drug effectiveness and toxicity is considered. Secondly, we did not investigate the effect of propofol and high glucose on mononuclear. So further experiments are required to determine whether the effect of propofol on high glucose-induced mononuclear-endothelial interaction is mediated by endothelial cells, mononuclear cells, or both.

## Conclusions

In summary, the present study suggested that 15 mM glucose, by inducing O_2_^.-^ accumulation, PKCβ2 Ser^660^ phosphorylation and PKC activation, NF-κB activation, up-regulated endothelial adhesion molecules expression and augmented mononuclear-endothelial interaction. More importantly, our study demonstrated that propofol, via decreasing O_2_^.-^ accumulation, down-regulating PKCβ2 Ser^660^ phosphorylation and PKC activity, inhibiting NF-κB activation, attenuated 15 mM glucose-induced endothelial adhesion molecules expression and mononuclear-endothelial adhesion. Our data implied the potential advantage of the administration of propofol to provide sedation in hyperglycemic patients in operation room or in post anesthesia care unit.

## Abbreviations

E-selectin: Endothelial selectin; ICAM-1: Intercellular adhesion molecule 1; VCAM-1: Vascular cell adhesion molecule 1; NF-κB: Transcription factor nuclear factor kappa B; HUVECs: Human umbilical vein endothelial cells; IκBα: Inhibitory subunit of NF-κBα; PKC: Protein kinase C; ROS: Reactive oxygen species; O_2_^.-^: Superoxide anion.

## Competing interests

The authors declare no conflicts of interest.

## Authors’ contributions

MZ conducted the experiments and contributed to the study implementation, statistical analysis, interpretation, and the preparation of the manuscript. JC conducted the experiments and contributed to the study design, implementation, and the preparation of the manuscript. Both MZ and JC contributed equally to this paper. HJ helped to conduct the experiments. CM supervised the study conduction and contributed to the study design, implementation, statistical interpretation, the preparation and finalization of the manuscript. All authors approved the final manuscript for publication.
